# Supplementary material for: Rapid plastid isolation reveals the chloroplast proteome and structures of the chlororibosome large subunit and RuBisCO in Marchantia polymorpha
Source: bioRxiv. 2026 Jul 20:2026.07.14.738477. Preprint. [Version 2] doi: 10.64898/2026.07.14.738477 (PMC13419835; doi:10.64898/2026.07.14.738477)
Supplement: Supplementary file 1 [file media-1.pdf]

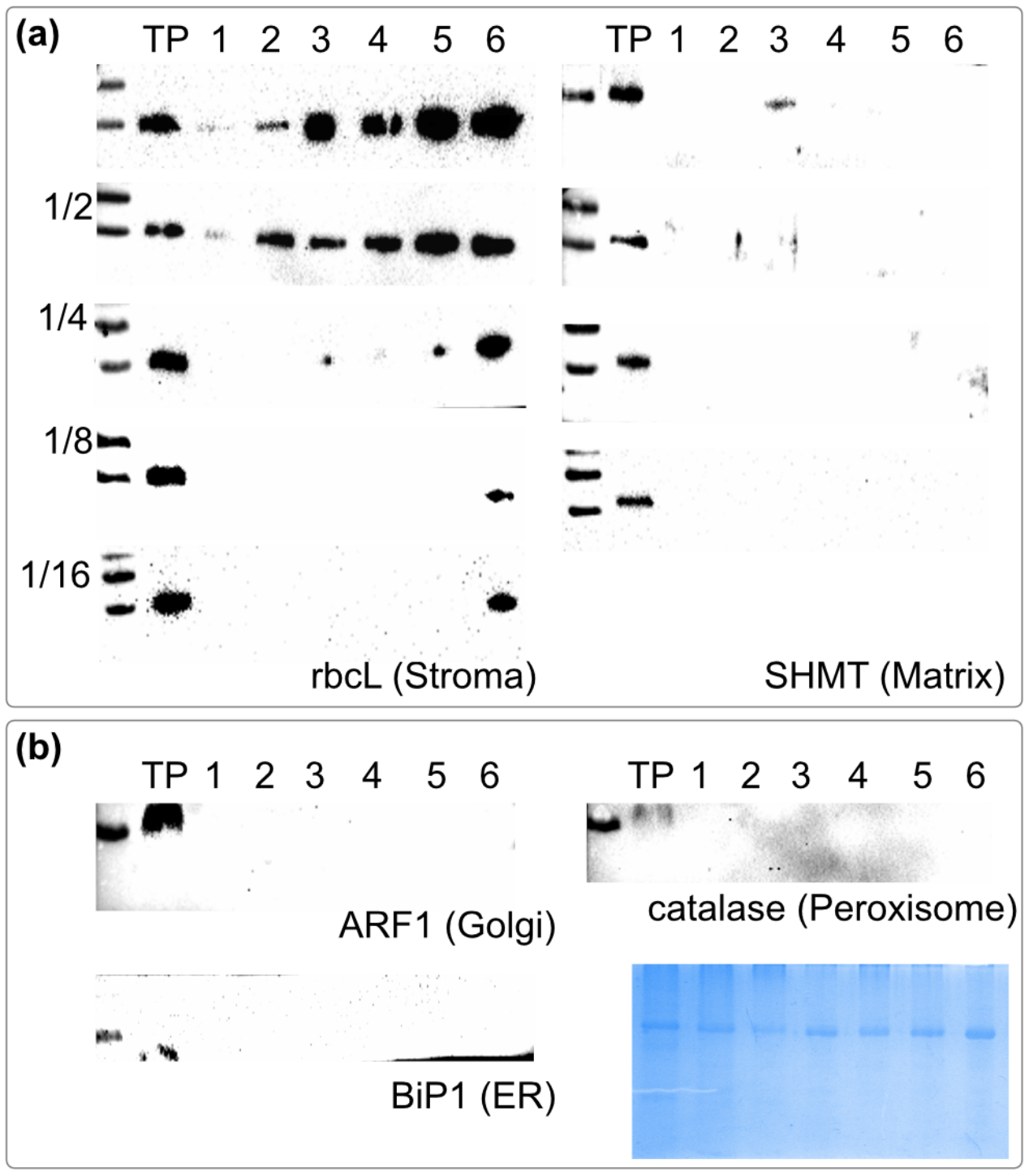

Figure S1: Enrichment of plastids from *Marchantia*. (a) Immunoblotting from total protein (TP) and chloroplast protein from six independent chloroplast preparations from ca. 10 g (Prep 1-2), 20 g (Prep 3-4) and 30 g (Prep 5-6) thalli against rbcL (stroma marker) and SHMT (mitochondrial marker). The plastid proteins serially diluted from 1/2 to 1/16 underscore the enrichment of the isolated plastids. **(b)** Immunoblotting against marker proteins for the Golgi, ER, peroxisome and Coomassie-staining showing even sample loading. A prestained protein ladder is loaded in the first well of each gel.

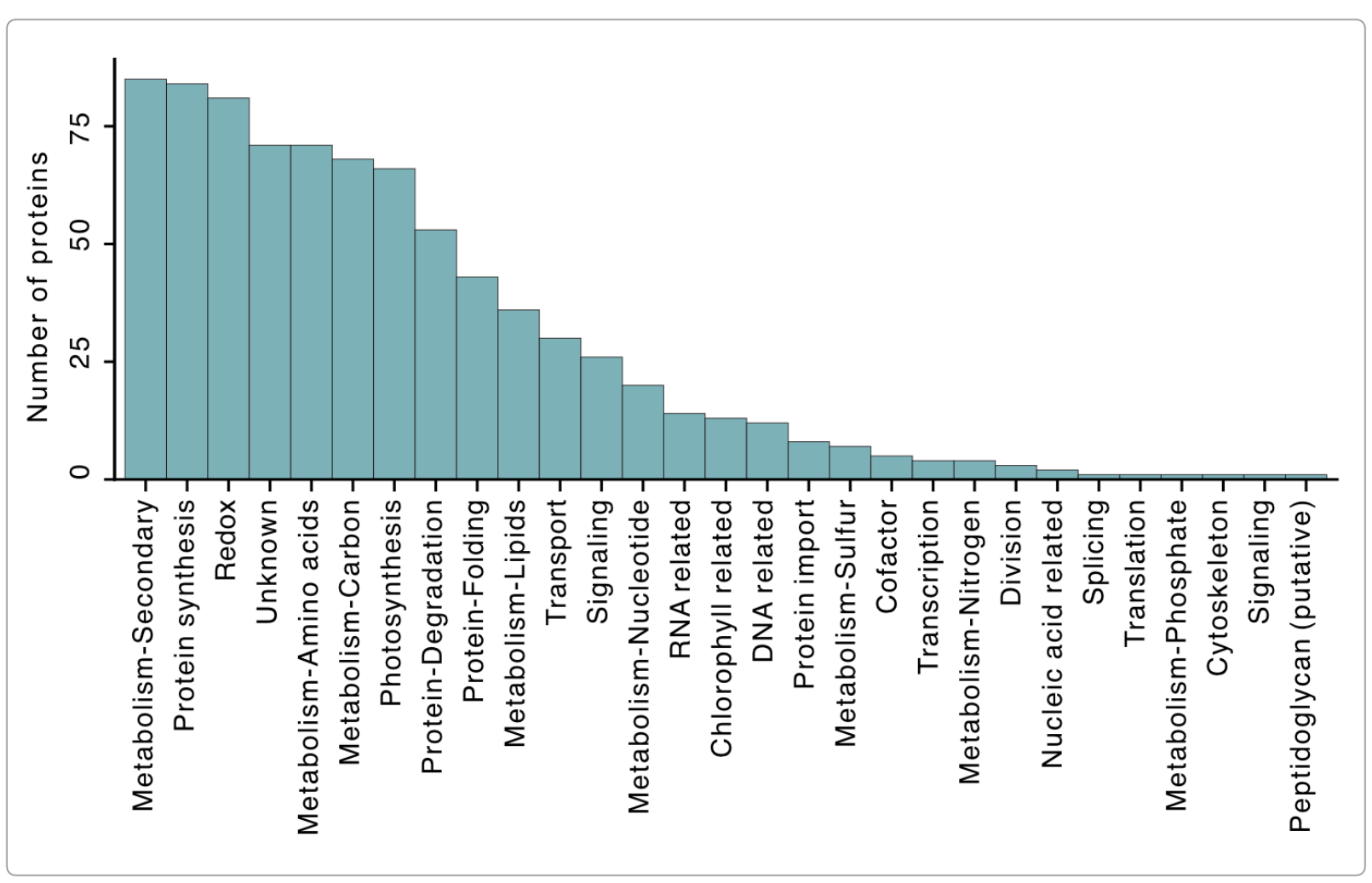

Figure S2: Cyanobacterial homologues of *M. polymorpha* plastid proteins. Functions of 812 *M. polymorpha* plastid proteins with homologues in a database derived from 56 cyanobacteria. Another 527 *M. polymorpha* plastid proteins lack homologues in Cyanobacteria.

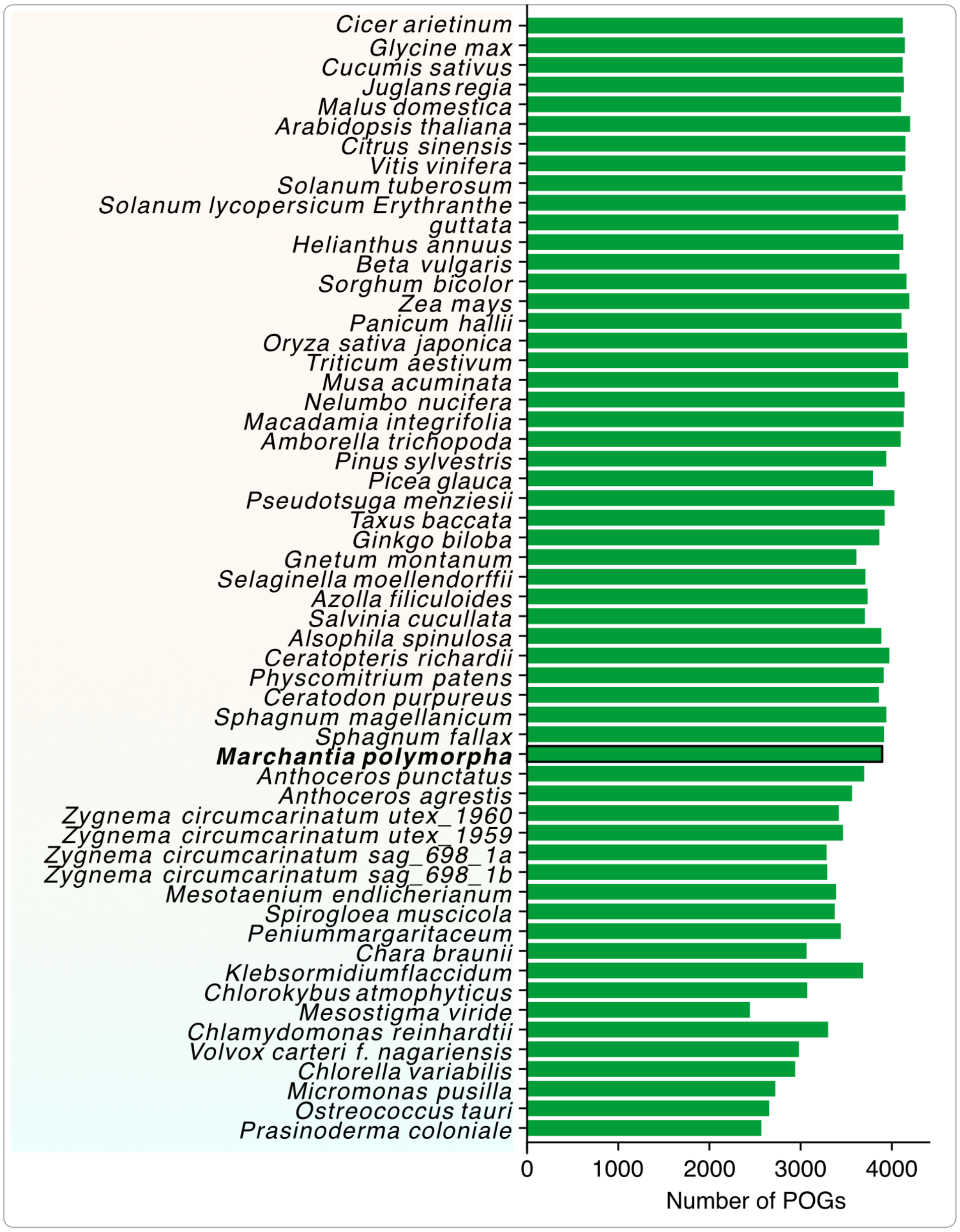

Figure S3: Number of plastid localized orthogroups per species across 57 diverse chloroplastida species.

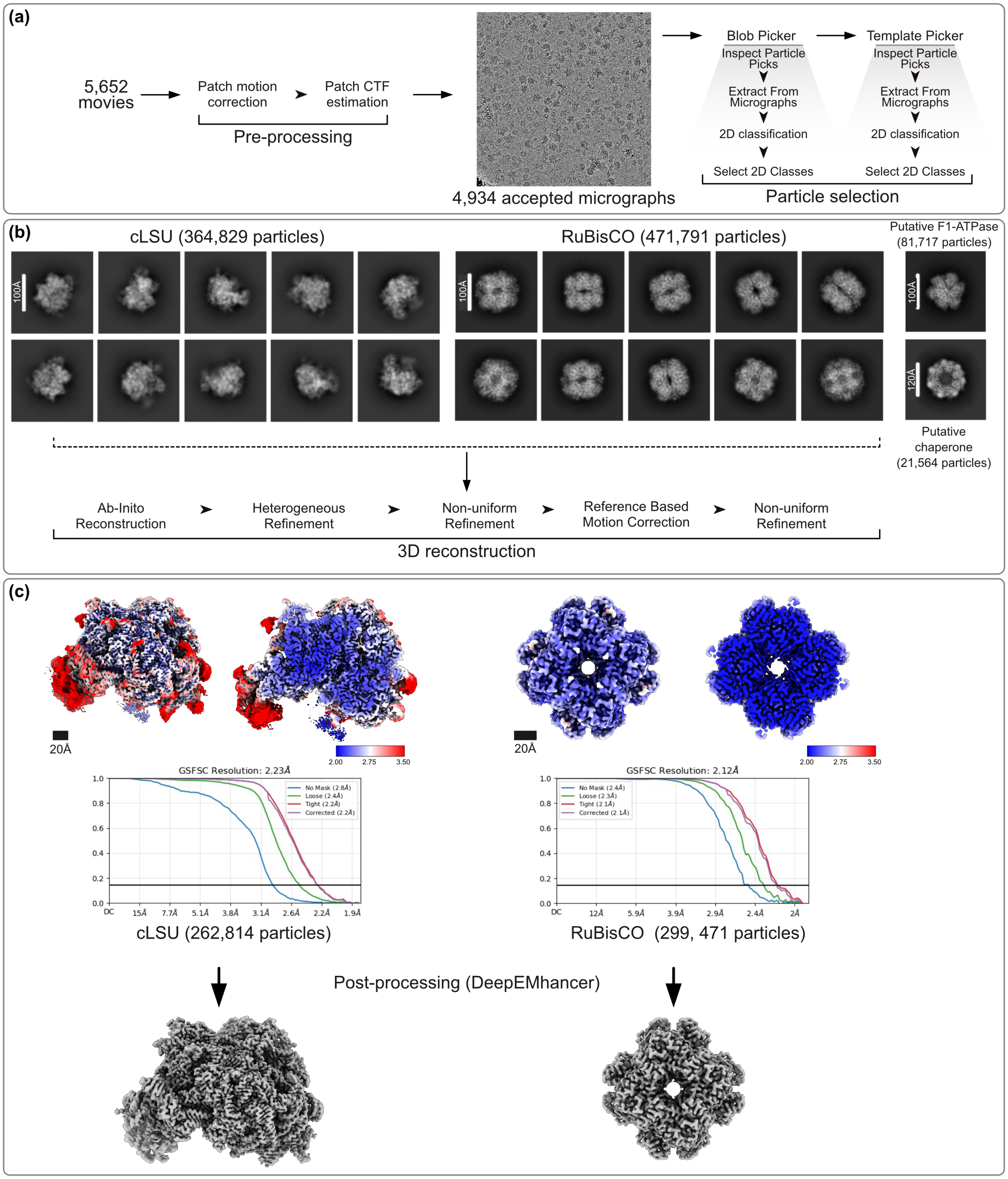

Figure S4: CryoSPARC processing workflow and exemplary results. (a) 5,652 cryoEM movies were preprocessed, resulting in 4,934 micrographs meeting the quality thresholds. Particles were selected from these micrographs via Blob Picker and Template Pickers, with a micrograph shown. **(b)** The workflow resulted in ca. 300-400K particles for chloroplast large subunit (cLSU) and RuBisCO, alongside <100K particles of putative F1-ATPase and Chaperone, with example 2D class averages shown. From the cLSU and RuBisCO particles, 3D images were reconstructed at high resolution, with the local resolution maps

(left: surface, right: central cross section) and FSC curves shown. **(c)** Density maps were sharpened via DeepEMhancer, and the resulting sharpened maps are shown.

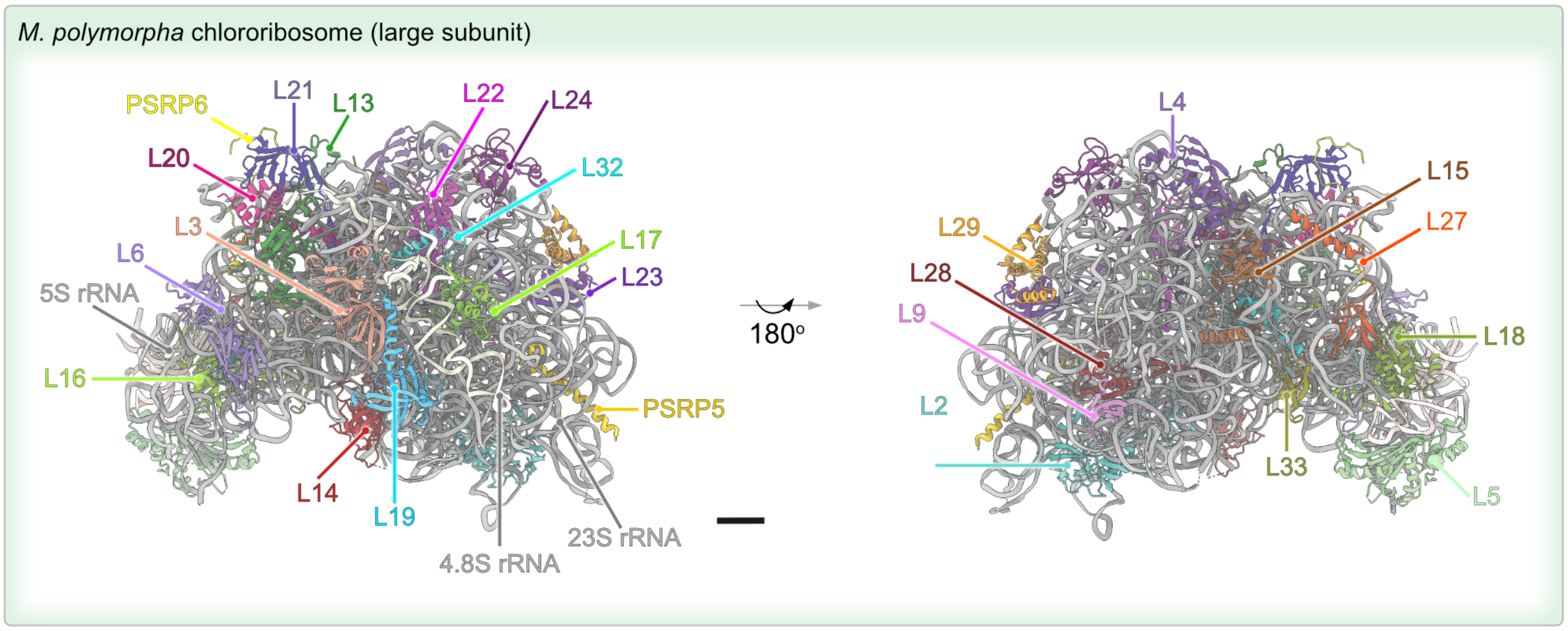

Figure S5: Structure of Marchantia chlororibosomal large subunit. Atomic model of *M. polymorpha* cLSU, as ribbon representation, colored according to individual RPs and rRNAs**.**

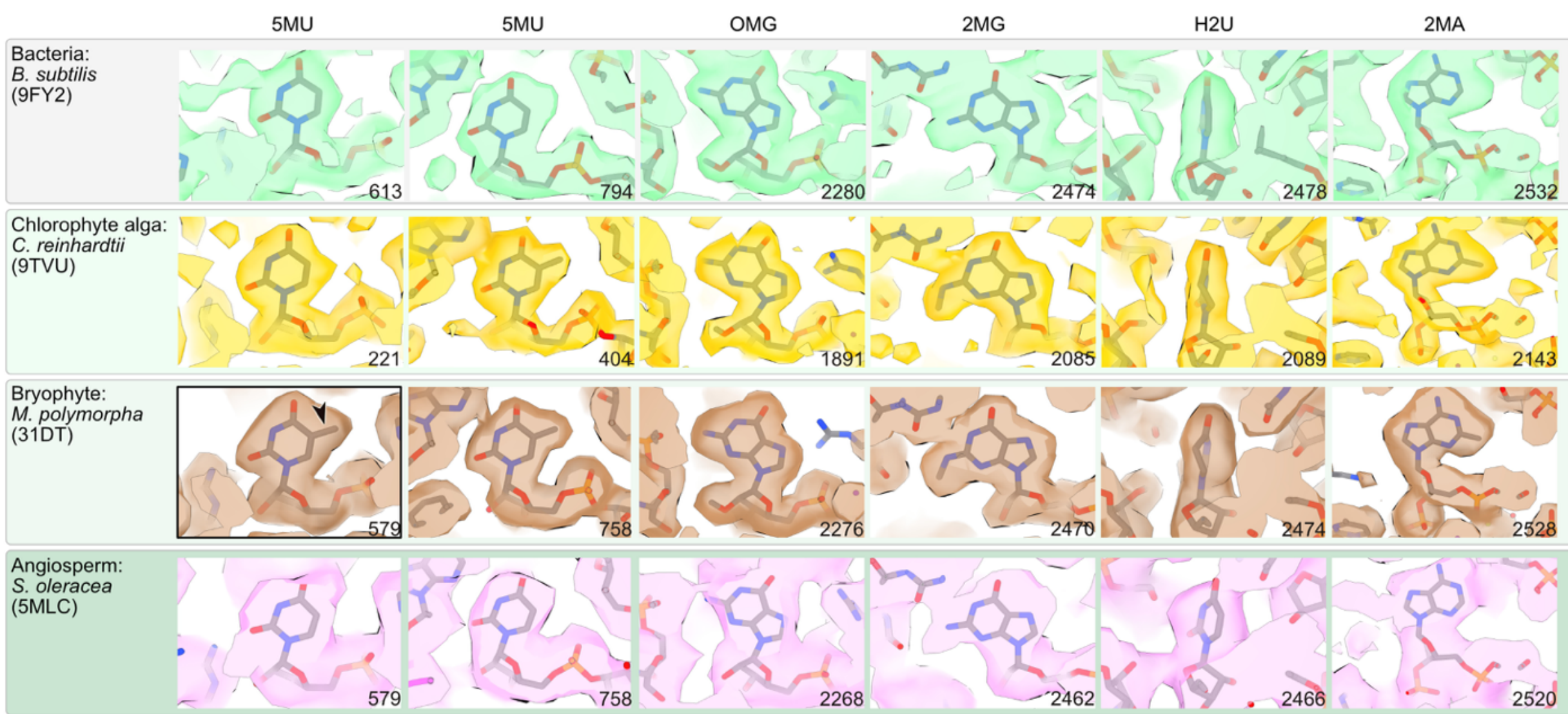

Figure S6: rRNA modification from chlororibosomes across species. Modifications in the 23S rRNA of chlororibosomes with the bases labeled on the bottom right in each image. A modification likely unique to *M. polymorpha* is indicated by a black border and an arrow within the image. (5MU = 5-methyluridine, OMG = 2′-O-methylguanosine, 2MG = 2N-methylguanosine, H2U = 5,6-dihydrouridine, 2MA = 2-methyladenosine).

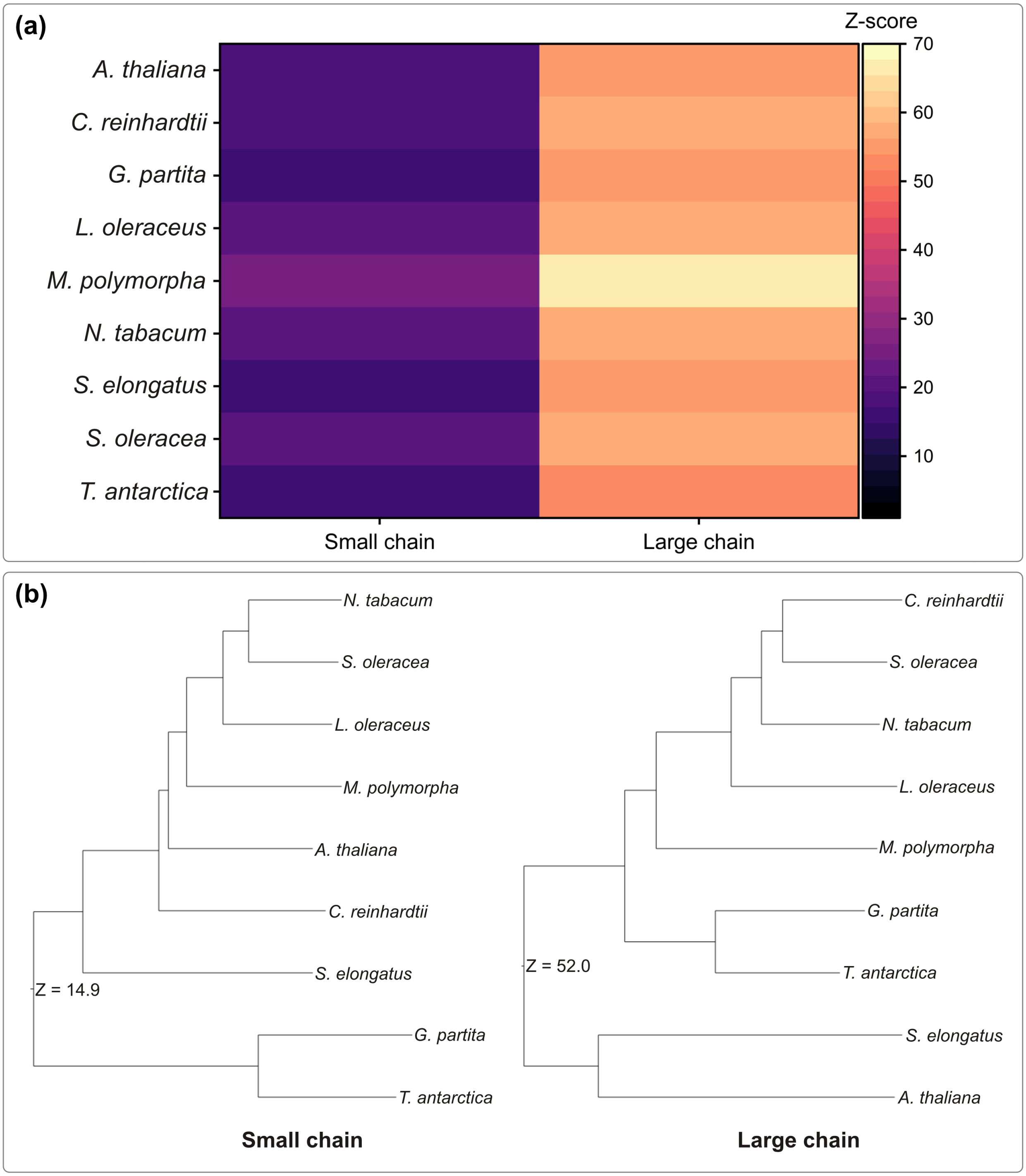

Figure S7: Structural comparison of RuBisCO across species. DALI Z-score across nine species represented as a heatmap **(a)** and cladograms **(b)**. (*A. thaliana* = *Arabidopsis thaliana, C. reinhardtii* = *Chlamydomonas reinhardtii, G. partita* = *Glaucocystis partita, L. oleraceus* = *Lophomonas oleraceus, M. polymorpha* = *Marchantia polymorpha, N. tabacum* = *Nicotiana tabacum, S. elongatus* = *Synechococcus elongatus, S. oleracea* = *Spinacia oleracea, T. antarctica* = *Trebouxia antarctica*). Compared structures and their respective PDB IDs are referred to in Fig. 5.
